# Genome-Wide Scan Identifies Loci Associated with Classical BSE Occurrence

**DOI:** 10.1371/journal.pone.0026819

**Published:** 2011-11-04

**Authors:** Brenda M. Murdoch, Gordon K. Murdoch, Matthew Settles, Stephanie McKay, John L. Williams, Stephen S. Moore

**Affiliations:** 1 Department of Agricultural, Food and Nutritional Science, University of Alberta, Edmonton, Alberta, Canada; 2 School of Molecular Biosciences, Washington State University, Pullman, Washington, United States of America; 3 Animal and Veterinary Science, University of Idaho, Moscow, Idaho, United States of America; 4 Department of Biological Sciences, University of Idaho, Moscow, Idaho, United States of America; 5 Division of Animal Sciences, University of Missouri, Columbia, Missouri, United States of America; 6 Parco Tecnologico Padano, Polo Universitario, Lodi, Italy; Institut Pasteur, France

## Abstract

Classical bovine spongiform encephalopathy (BSE) is an acquired prion disease that is invariably fatal in cattle and has been implicated as a significant human health risk. Sequence variations in the coding region of the prion gene (*PRNP*) have been associated with acquired transmissible spongiform encephalopathy (TSE) susceptibility in mammals; however, this is not the case in cattle. It has been hypothesized that genes, in addition to the prion gene, contribute to genetic susceptibility of acquired TSEs. Accordingly, genetic studies of classical BSE in cattle identified loci other than *PRNP* that are associated with disease incidence. The objective of this study was to utilize a genome-wide association study to test for genetic loci associated with classical BSE. The samples include 143 BSE affected (case) and 173 unaffected half sib (control) animals collected in the mid 1990s in Southern England. The data analysis identifies loci on two different chromosomes associated with BSE disease occurrence. Most notable is a single nucleotide polymorphism on chromosome 1 at 29.15 Mb that is associated with BSE disease (p = 3.09E-05). Additionally, a locus on chromosome 14, within a cluster of SNPs showed a trend toward significance (p = 5.24E-05). It is worth noting that in a human vCJD study markers on human chromosome 8, a region with shared synteny to the region identified on cattle chromosome 14, were associated with disease. Further, our candidate genes appear to have plausible biological relevance with the known etiology of TSE disease. One of the candidate genes is hypothetical gene LOC521010, similar to FK506 binding protein 2 located on chromosome 1 at 29.32 Mb. This gene encodes a protein that is a member of the immunophilin protein family and is involved in basic cellular processes including protein folding. The chromosomal regions identified in this study and candidate genes within these regions merit further investigation.

## Introduction

Transmissible spongiform encephalopathies (TSEs), also known as prion diseases, are a group of invariably fatal mammalian neurodegenerative diseases [1]. TSEs are unique in that they can manifest through inherited, sporadic or acquired origins [2]. Classical bovine spongiform encephalopathy (cBSE) is an acquired TSE that is transmissible through the consumption of meat and/or bone meal contaminated with the infectious prion agent. Dietary exposure to food contaminated with the BSE agent is the suspected primary cause of the human TSE, variant Creutzfeldt-Jakob Disease (vCJD) [3]–[6].

The prion protein is a glycosyl-phosphatidylinositol (GPI) anchored protein that has a native form (PrP^c^) for which the secondary structure consists predominantly of alpha-helices. The disease-associated form, PrP^Res^, has a substantial increase in the beta-pleated sheet content and reduction of the alpha-helices in comparison to the native form [7]. The presence of PrP^Res^ promotes the conversion of native PrP^c^ to PrP^Res^ via a mechanism that is to date not completely understood [8]. This altered protein conformation is associated with an increased resistance to digestion by proteinase K [9]. As a consequence of this resistance to protein degradation, the accumulation of PrP^Res^ within neurons results in the characteristic plaques that are part of the etiology of prion disease.

Moreover, the accumulation of abnormally folded prion protein within the central nervous system mediates a conformational change in the native host protein to a protease-resistant form of the prion protein [1], [2]. The central nervous system (CNS) is the primary tissue where the disease form of the protein accumulates. However, in the case of acquired TSEs the infectious agent is thought to be transmitted through the Peyer's patches in the intestine, to the CNS [10].

Expression of the prion protein is essential for the development of TSEs [11], and sequence variations in the prion gene (*PRNP*) have been associated with TSE susceptibility in humans, mice, sheep, deer and to a lesser extent cattle [10]. Specific polymorphisms in the coding region of *PRNP* in humans and sheep are associated with acquired TSE susceptibility [12]–[14]. In general this is not the case in cattle, although alleles and haplotypes containing insertion/deletion polymorphisms in the bovine *PRNP* promoter region have been associated with incidence of classical bovine spongiform encephalopathy (BSE) [15]–[19]. It has been hypothesized that genes, in addition to the prion gene, contribute to genetic susceptibility of acquired TSEs. Genome-wide studies in sheep [20]–[22], mice [23]–[25], and humans [26] have identified chromosomal regions, other than the prion gene, that contribute to TSE disease progression. Furthermore, genetic studies of classical BSE in cattle identified loci other than *PRNP* (located on chromosome 13 at 47.2 Mb) that are associated with disease incidence [27]–[29].

The studies that have been performed with cattle thus far use relatively low density panels of either microsatellite or SNP markers. With recent advancements in sequencing and SNP marker technology it is possible to perform an association analysis with greater resolution to those previously described. As a result the current genome-wide association study allows insight into two important questions. The first is whether an analysis with a higher resolution scan identify novel genomic regions associated with classical BSE. Secondly, whether such an analysis can confirm and/or refine the previously identified genomic regions and ultimately identify positional candidate genes.

The objective of this study was to utilize a high-density whole genome association study to identify genetic loci associated with classical BSE. The animals used are female Holstein cattle from the UK, that contracted BSE in the mid- 1990's, along with matched half-sib controls. The BSE cases were identified on commercial farms where the most likely source of disease was through consumption of contaminated feed prior to and during the BSE epidemic in the United Kingdom. The BSE cases were paired with age matched half-sib controls from the same calving season and from the same farm and samples were used in case-control association analysis.

## Materials and Methods

### Animal Information

**Figure 1:**
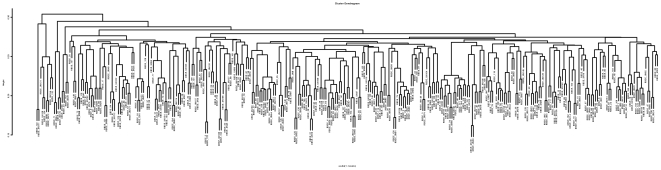
Dendrogram showing the relationship of BSE positive (case) and control samples. Derived from genotypic data use R 2.9.0. cluster diagram statisical software.

This study used DNA extracted from blood samples of Holstein cows that were diagnosed as BSE positive and half-sib controls collected in the mid 1990s in Southern England. The samples included 143 BSE affected (case) and 173 unaffected (control) animals. In addition 15 BSE negative animals, defined by post-mortem histology, were included in the control set. BSE positive cattle were examined by qualified veterinarians and their BSE status was subsequently confirmed post-mortem by histology (by the Veterinary Laboratories Agency, New Haw, Surrey, UK). Control animals were contemporary age matched half siblings (the same sire and different dams) of BSE cases, from the same calving season and farm and were healthy at the time samples were collected. As such, the control animals are assumed to have been exposed to the same environment. The dendrogram showing relationships among the samples is shown in Figure 1.

### Genotyping and Data quality control

The 316 BSE case and control samples were genotyped with a Illumina bovine SNP50 beadchip with more than 58k SNPs following the manufacturers recommended protocol (Illumina Inc., San Diego, CA). Genotypes were called using the BeadStudio software (Illumina Inc.) and processed through the automated genotype calling. Genotypes were then subjected to data quality control parameters.

For the case-control sample set from the original 58,336 SNPs in the assay, 1389 SNPs were excluded based on assay failure, as observed by poor clustering of alleles using the BeadStudio software. The remaining 56,947 SNPs were analyzed for quality control through the summary statistics of the PLINK program [30] and 2,065 SNPs were removed because more than 10% of the samples failed to genotype at that locus (GENO>0.1). Additionally, 5 samples were removed due to low genotyping rate (MIND>0.1). A further 8,739 SNPs were removed due to a minor allele frequency (MAF)<0.01. The remaining individuals that were included in the analyses had a mean genotyping rate of 0.97. Of these, an average 48,053 passed quality control measures and had an average genotyping success rate of 0.97.

**Figure 2:**
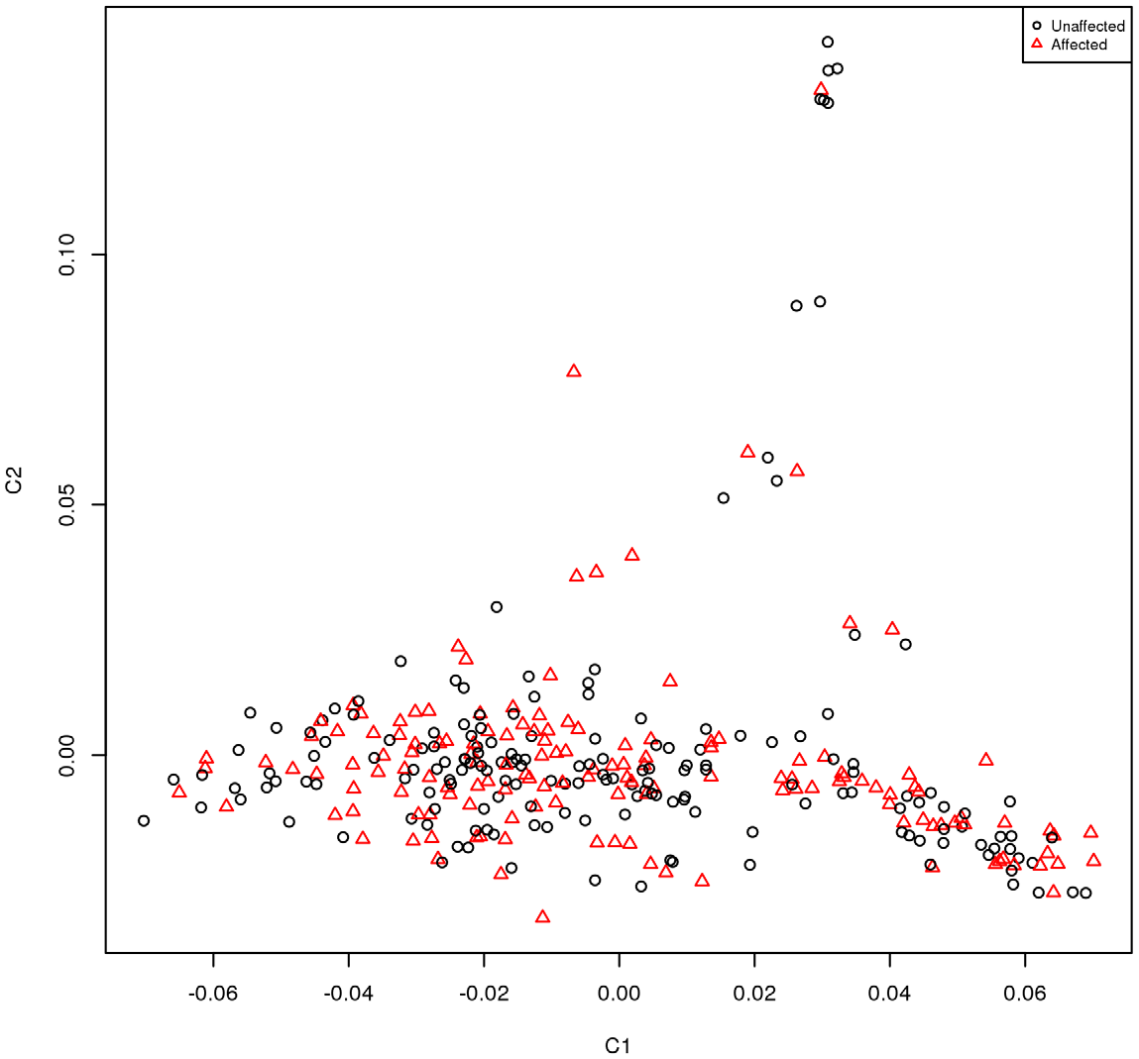
Multi-dimensional Scaling (MDS) of the BSE case and half-sib control set sample set.

Multi-dimensional scaling (MDS) of the matrix of genome-wide identity-by-state (IBS) distances was used to provide a two-dimensional projection of the data onto axes representing components of genetic variation. Animals whose genetic ancestry differs significantly appear as outliers on the MDS plot. The genome-wide IBS pair-wise identities between each pair of animals were calculated using PLINK [30]. These IBS-relationships were converted into genetic distances by subtracting them from one, and the matrix of pair-wise IBS distances was used as input for multi-dimensional scaling. Thus population stratification was tested for in this data set and none was observed (Figure 2).

### Statistical analysis

The PLINK software v1.04 was used to perform the statistical analysis [30]. The genotypic data from the BSE case and control sample set were analyzed using the case-control association (χ^2^) test. Additionally using the max (T) permutation within PLINK software, the data was subjected to 1,000,000 permutations and the p-values reported. Further to the allelic association test, associations were analyzed with the dominant and the recessive modes of inheritance all of which employed one degree of freedom (d.f.). Uncorrected p-values of 5E-5 were considered moderate evidence of statistical significance and this is further supported by the permuted p-values. All of the plots and figures were produced using R statistical analyses. Physical positions and alleles are expressed in terms of the forward strand of the reference genome (BTAU4.0, ftp://ftp.hgsc.bcm.tmc.edu/pub/data/Btaurus/).

## Results

### Case-control association analysis for BSE Incidence

The case-control samples were comprised of 143 BSE case and 173 control animals. The control samples included at least one animal from the same farm as each of the BSE cases and an additional 15 BSE negative animals. All samples were genotyped with the bovine SNP50 beadchip that consisted of greater than 58K SNPs dispersed across the genome with an average interval of 37 kb [31]. Of these markers, an average 48,053 SNPs passed quality control measures outlined in the material and method section with an average genotyping success rate of 0.97. These data were analyzed for association with disease status using the case-control allelic test within the PLINK software [30].

**Figure 3:**
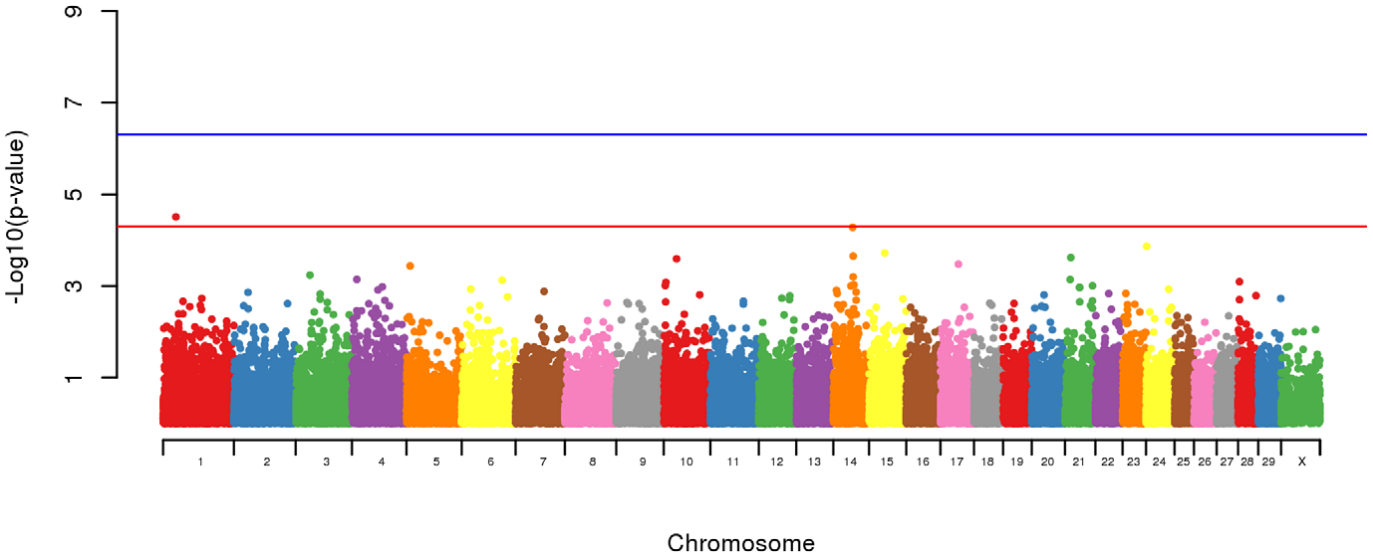
Genome-wide plot of −log10 p-values of loci from BSE case-control allelic association analysis. Bovine autosomes 1–29 and Chromosome X are shown by different colors. The horizontal lines show moderate (5E-5) and high (5E-7) significance thresholds.

An allelic association analysis of the 316 animal across 48,053 SNPs revealed 1 SNP on chromosome 1 at 29,147,078 bp with a significant (p = 3.09E-5) over representation in BSE affected animals. Possibly of greater interest was a locus on chromosome 14 where, although the peak SNP at 43,984,235 bp did not research significance (p = 5.24E-05) it was within a cluster of SNPs above background (see Table 1 and Figure 3). Additionally markers on chromosomes 5, 10, 14, 15, 17, 21 and 24 had P values approaching significance (p≤5E-4).

**Table 1:** Allelic association analysis of 50 K SNPs with BSE occurrence.

CHR	Position (Mb)	SNP ID	A1	A2	F-A	F-U	Unadjusted *p*-value	OR	Permuted *p*-value
1	29.1	BTB-01333940	A	G	0.15	0.05	3.09E-05*	3.225	3.70E-05
5	8.4	BFGL-NGS-117339	G	A	0.28	0.42	3.66E-04	0.528	4.23E-03
10	29.2	BTB-01261410	A	C	0.52	0.38	2.55E-04	1.808	1.72E-04
14	44.0	Hapmap57707-rs29024913	A	G	0.48	0.32	5.24E-05	1.945	5.40E-05
14	45.2	ARS-BFGL-BAC-23887	G	A	0.55	0.40	2.23E-04	1.812	1.20E-04
15	35.7	ARS-BFGL-NGS-65959	A	G	0.38	0.24	1.91E-04	1.914	1.08E-04
17	41.0	Hapmap43651-BTA-67415	A	G	0.31	0.45	3.34E-04	0.550	5.20E-04
21	13.2	Hapmap42965-BTA-53492	G	A	0.53	0.38	2.40E-04	1.810	1.44E-04
24	1.5	ARS-BFGL-NGS-11247	C	A	0.20	0.34	1.36E-04	0.494	2.55E-04

A1 is the major allele and A2 is the minor allele. The frequency of the major allele in the BSE affected (F-A), and unaffected (F-U) animals. Allelic association unadjusted *p*-value and OR is the Odds Ratio and *p*-value after 1,000,000 permutation.

*denotes moderate significance.

The data set was analyzed with a best fit model test in which standard allelic, trend, dominant, recessive and genotypic association tests were performed. The test result with the lowest p value is reported in Table 2. In addition to the loci already identified on chromosome 1 and 14, the best fit model identified three SNPs with recessive and two SNPs with dominance effects. Specifically, SNP on chromosomes 2, 3 and 21 exhibited suggestive associations with BSE under a recessive model while loci on chromosomes 14 and 24 exhibited suggestive associations under a dominance model. The locus at 45,169,821 bp on chromosome 14 (associated with the dominance model) is in close proximity (∼1.2 Mb away) to the loci identified in the allelic association (see Table 2).

**Table 2:** Model association analysis of BSE occurrence with 50 K SNPs.

CHR	Position (Mb)	SNP ID	A1	A2	TEST	*p*-value
1	29.1	BTB-01333940	A	G	ALLELIC	3.09E-05*
2	108.6	BTA-48618-no-rs	G	A	REC	9.10E-05
3	32.4	ARS-BFGL-NGS-104661	A	C	REC	5.87E-05
14	44.0	Hapmap57707-rs29024913	A	G	ALLELIC	5.24E-05
14	45.2	ARS-BFGL-BAC-23887	G	A	DOM	6.44E-05
21	13.2	Hapmap42965-BTA-53492	G	A	REC	9.30E-05
24	1.5	ARS-BFGL-NGS-11247	C	A	DOM	6.45E-05

Duplicate loci were removed and unadjusted *p*-values are reported.

*denotes moderate significance.

## Discussion

### Classical BSE

Classical BSE disease is a difficult phenotype to define. Animals that have developed BSE are clearly susceptible. However, those appearing clinically healthy may either be incubating disease though clinically asymptomatic, may be in fact resistant to disease, or may not have been sufficiently exposed to the infectious agent. Alternatively, they may have been less susceptible to the disease through resistance or requirement for higher infectious dose for acquisition of disease. Because disease is thought to occur following migration of the infectious agent from the intestine to the brain, multiple genes are likely to be involved in the acquisition and transfer of the agent as well as progression to disease. Consequently, the development of classical BSE may involve the interaction and possibly subtle effects of several contributing loci and is likely to be a complex trait involving genes expressed differentially in several tissues. Therefore, it is important to consider this when examining and interpreting the results for evidence of associations between disease and genetic loci. Specifically, the balance of applying stringent multiple testing corrections, such as Bonferroni, to a phenotype such as this may be overly prone to type II errors and discarding real, though subtle associations versus a type I errors that generates false positives. Additionally, because this case control analysis used samples from naturally infected cattle during the UK epidemic the number of contemporaneous controls was limited and thus limits the power of this study. The power of this study has a range from 0.58 to 0.85 depending on the unknown probability of exposure in the control animals. A larger number of samples would have increased the power to detect significant loci, however this study utilized the largest number of samples available to us.

### Associations with classical BSE disease presence or absence

The higher resolution genomic analyses utilized in this study have identified loci on several different chromosomes that are associated with BSE disease status. Most notable is a single nucleotide polymorphism on chromosome 1 at 29.15 Mb that is significantly (p = 3.09E-05) associated with BSE disease, suggesting that this is functionally pertinent to risk of disease acquisition and/or progression. Similarly, another locus, on chromosome 14 at 32.4 Mb, (p = 5.24E-05), is identified to be associated with BSE disease. The results on chromosome 14 are further supported by a SNP 1.2 Mb away that is also associated both in a dominance model (6.44E-05) and in an allelic model (2.23E-04) with disease. Additionally, there is evidence of associations with absence of disease in control animals on two chromosomes. A locus on chromosome 3 at 32.4 Mb was associated (p = 5.87E-05) with the absence of disease under a recessive model, whereas a locus on chromosome 24 showed an association (p = 6.45E-05) under a dominance model.

### Regions identified in previous analysis and studies in Holstein cattle

Samples used in this study overlap with a subset of those in our previous study [29], and therefore the results are not completely independent. However, the most convincing evidence that previously reported associations are valid is the fact that they were again identified in this higher resolution genomic analysis and with a different sample set to previous studies [27], [28]. The locus on chromosome 1 was the most significant in the present study. An association on this chromosome was also identified by Zhang et al. [28] in a low density QTL study, and although the position of the peak LOD score in that study was at the other end of the chromosome the SNP identified here was within the 95% confidence interval. The region identified in this study on chromosome 14 was also previously reported [29], using the same samples but with a different set of SNPs and as such this represents a technical verification of the earlier data. It is worth noting, however, that in a study of genetic control of CJD in humans, markers on human chromosome 8 that share conservation of synteny with the region of cattle chromosome 14 were identified [26]. With the exception of the locus on chromosome 3, which has not been previously reported as associated with BSE, all other associations identified in this study provide confirmation of associations identified in previous studies using a lower density and different markers [27]–[29] (see Table 3). This study, like others [22]–[25], indicates the presence of multiple loci on a number of chromosomes that may have subtle effects on BSE susceptibility.

**Table 3:** Comparison of loci identified in this study with those previously reported.

CHR	Position Mb	SNP	*p-value*	Previously observed	Study	Location Mb
1	29.1	BTB-01333940	3.09E-05	Yes	[28], [29]	89.7
2	108.6	BTA-48618-no-rs	9.10E-05	Yes	[29]	37.1
3	32.4	ARS-BFGL-NGS-104661	5.87E-05	no		
5	8.4	BFGL-NGS-117339	3.66E-04	Yes	[27], [29]	30.6
10	29.2	BTB-01261410	2.55E-04	Yes	[27], [29]	21.1
14	44.0	Hapmap57707-rs29024913	5.24E-05	Yes	[26], [29]	44.0
15	35.7	ARS-BFGL-NGS-65959	1.91E-04	Yes	[29]	35.7
17	41.0	Hapmap43651-BTA-67415	3.34E-04	Yes	[28], [29]	44.2
21	13.2	Hapmap42965-BTA-53492	9.30E-05	Yes	[29]	33.2
24	1.5	ARS-BFGL-NGS-11247	6.45E-05	Yes	[29]	18.5

### Candidate genes

In a 2006 review, Mabbott and MacPherson discussed how the identification of the route of oral transmission, from the site of infection to the brain, may facilitate the development of treatments for TSEs. This process would include, transport of the prion across the gut epithelium, transport to follicular dendritic cells, accumulation in lympheatic cells and tissues and progression from lymphatic tissues to tissues of the CNS including the brain [10]. To this end our study employed a genomic approach to elucidate chromosomal regions of association with disease or absence of disease. It is useful to characterize whether genes in the identified regions may reflect a physiological contribution to the transmission pathway of infectious prions from the gut to the brain. Some of our candidate genes appear to have attributed biological functions that may intuitively be associated with the known etiology of TSE disease, specifically the candidate gene, hypothetical gene LOC521010, similar to FK506 binding protein 2 located on chromosome 1 at 29,316,874 bp. This gene encodes a protein that is a member of the immunophilin protein family and is involved in basic cellular processes including protein folding. The encoded protein of this gene is a cis-trans prolyl isomerase, similar to cylclophilin A, and binds the immunosuppressants, FK506 and rapamycin. While the identification of an association of this genomic region with disease is far from a confirmation of a causative role, it is within logic to speculate how this gene may be related to the known parameters of TSE disease. It is well accepted that the pathology associated with TSEs includes the alteration of protein folding of the native PrP^c^ to a misfolded conformation PrP^Res^ and therefore the identification of this gene known to transcribe a protein involved in intracellular protein folding is at a minimum, compelling.

The SNP located on BTA 14 (43.9 Mb), associated with BSE disease case-control analysis, is in close proximity to the gene exostoses (multiple) 1, *EXT1*. McCormick et al. [32], showed that *EXT1* is an endoplasmic reticulum (ER)-resident type II transmembrane glycoprotein whose expression in cells results in the alteration of the synthesis and display of cell surface heparin sulfate glycosaminoglycans (GAGs). The N terminus of PrP contains a GAG-binding motif and it is thought that PrP binding of GAG is important in prion disease [33]. Again, if one considers that the PrP contains a binding motif for cell surface GAGs, then a gene such as exostoses may provide a means for PrP to adhere to gastrointestinal and neural cells, and thus this candidate gene may be important in the disease acquisition and progression.

Additional positional candidate genes of interest on chromosome 3 approaching significance include: leucine-rich repeats and immunoglobulin-like domains 2, LRIG2, a protein which participates in protein-protein interactions as well as Repulsive guidance molecule family member A, a glycosyl phosphatidylinositol-anchored glycoprotein that functions as an axon guidance protein in the central nervous system. The functions of the candidate genes identified in this study infer a role in the disease etiology especially those related to immunological tissue and neuronal tissue transport mechanisms.

Ideally it would be of interest to test if the associations identified in this study would replicate in an additional sample set. However, as these samples were collected during the UK BSE epidemic the availability of contemporaneous controls are limited. Regardless the validation of these associations in either another cattle population or species would be extremely useful. Additionally, it is important to emphasize that the candidate genes identified through this genomic association study neither confirm nor imply direct involvement of these genes in the disease process. Future studies directed at assessing these genes are necessary to identify any physiological relationships between their functions and the incidence of TSE disease.

### Conclusion

We [29] and others [9], [22]–[28] hypothesize the involvement of genes in addition to the prion gene in the progression of prion diseases. In particular work by Mead S. *et al.* 2009, used a genome-wide association study to identify genetic risk factors for vCJD where the genetic region that they identified comparatively overlaps that which we identified [26]. This high-density genome-wide association analysis identified a SNP on bovine chromosome 1 that was significantly associated with BSE disease. Moreover, the locus on chromosome 14 confirms an association identified in an earlier study [29] and at a region of conserved synteny with human chromosome 8 where an association has been reported with vCJD [26].

We report data that demonstrate an association of specific regions in cattle with classical BSE disease occurrence. Information regarding the involvement of genes, in addition to the prion gene, in the progression of prion disease is invaluable and merit further investigation. Not only will it enable us to gain a better understanding of the pathobiology of prion diseases but may also provide insight towards development of effective gene-specific treatments of Prion related diseases.
